# Epidemiological Characteristics of Mumps Cases in the Kingdom of Bahrain From 2012 to 2022

**DOI:** 10.7759/cureus.76551

**Published:** 2024-12-29

**Authors:** Ebrahim Matar, Afaf Merza Mohamed, Ghufran J Ali, Fatema Shaker Ali, Muna Husain, Ahmed K Moosa, Basma Mahmood Al Saffar, Adel S Alsayyad

**Affiliations:** 1 Public Health Directorate, Ministry of Health, Manama, BHR; 2 Department of Pediatrics, Salmaniya Medical Complex, Manama, BHR; 3 Department of Family and Community Medicine, Arabian Gulf University, Manama, BHR

**Keywords:** bahrain, mumps, mumps epidemiology, mumps outbreak, mumps vaccine

## Abstract

Introduction: Mumps is a vaccine-preventable disease caused by the paramyxovirus affecting the salivary gland and may be complicated by orchitis, oophoritis, and encephalitis. This study aims to describe the epidemiology, clinical presentation, and transmission of mumps cases in the Kingdom of Bahrain between 2012 and 2022.

Methodology: A retrospective cross-sectional study was conducted using national surveillance data of confirmed mumps cases, including all age groups and both Bahraini and non-Bahraini nationals, from January 2012 to December 2022.

Results: A total of 258 cases were recorded, with the majority being male (159, 61.6%). Most patients (255, 98.8%) presented with parotid gland swelling, and 9 (3.5%) required hospitalization. Only 63 (24%) patients had documented vaccination history. Incidence rates varied, with peaks in 2013, 2015, and 2019. The highest incidence was observed in children aged 0-4 years in 2012, shifting to the 5-14 years age group from 2013 to 2015, and then returning to the 0-4 years age group from 2016 to 2022. Overall, there is a downward trend in mumps cases in Bahrain.

Conclusions: The study shows a declining trend in mumps incidence in Bahrain. The high incidence in young children emphasizes the need to maintain high vaccination coverage. Enhanced surveillance, timely outbreak investigations, interventions, and public health vaccine campaigns are essential to sustain progress. This comprehensive analysis provides baseline data and a foundation for strengthening mumps prevention and control efforts in Bahrain.

## Introduction

Mumps is a common contagious infection mainly affecting the pediatric age group. It is one of the vaccine-preventable diseases. It is caused by paramyxovirus, a member of the Rubulavirus family [1]. Parotid gland enlargement is a cardinal sign of infection. Mumps can be complicated by orchitis and oophoritis, which can develop in adult men and women, respectively, as well as encephalitis, aseptic meningitis, and pancreatitis [1]. While unilateral and bilateral parotid swelling aids in clinical diagnosis, it is not exclusively indicative of mumps [1]. Laboratory diagnosis involves virus isolation, viral nucleic acid detection, or serological confirmation, typically through the presence of immunoglobulin (Ig) M mumps antibodies [1].

The incubation period for mumps typically ranges from 16 to 18 days, but it can vary between 12 and 25 days. Mumps cases often begin with nonspecific symptoms such as fever and body aches. The hallmark sign of mumps is parotitis, which can be unilateral or bilateral and may involve other salivary glands. The swelling usually lasts around five days. In some cases, mumps may present only with non-specific symptoms, including respiratory symptoms. Approximately 15% to 24% of patients can be asymptomatic [2]. Infected individuals are recommended to isolate themselves for five days after the onset of symptoms to prevent the transmission of the disease to other people [3,4].

By 2023, mumps vaccine was introduced in 124 states [5]. A single dose of the mumps vaccination is approximately 80% effective in preventing the illness [1]. The introduction of the vaccine led to major changes in the epidemiology of mumps, with the incidence rates decreasing by up to 97% [6]. However globally, in the post-vaccine era, several countries, including the United States, the United Kingdom, Australia, and South Korea have experienced mumps outbreaks [7-11]. In the Middle East, the Kingdom of Saudi Arabia reported an outbreak, and an outbreak was also reported in the West Bank in the 2000s [12,13]. The resurgence of these outbreaks has been attributed to waning immunity, differences in vaccine strains, and the presence of vaccine-derived variants and wild-type virus strains, all of which require further investigation [7,14].

The Kingdom of Bahrain is an archipelago situated approximately 24 km off the east coast of Saudi Arabia, with a total area of 786.5 km^2 ^and a population of around 1.5 million [15]. The Ministry of Health provides preventive healthcare services to all mothers and children, including routine immunization [16]. The mumps vaccine is administered as part of the MMR (Measles, Mumps, and Rubella) vaccine, with two doses given at 12 and 18 months of age [17]. Since 2001, vaccine coverage has remained above 97% [18]. In Bahrain, mumps is classified as a Group A disease, requiring all suspected cases to be reported to the Public Health Directorate within 24 hours [19]. This study aims to analyze the national surveillance data to assess the epidemiology, clinical presentation, and trend of mumps in Bahrain from 2012 to 2022.

## Materials and methods

Study design and study setting

A retrospective cross-sectional study was conducted on all cases diagnosed with mumps and reported to the public health department in the Kingdom of Bahrain from 2012 to 2022.

Study population

The study included all confirmed mumps cases, that were reported to the Public Health Directorate, Kingdom of Bahrain, from 2012 to 2022, including all age groups, both sexes, and all nationalities (Bahraini and non-Bahraini nationals). According to Bahrain’s latest communicable diseases surveillance guideline, confirmed cases of mumps were defined as either laboratory-confirmed cases (through the presence of mumps-specific IgM antibodies, a fourfold or greater increase in mumps-specific IgG antibody titer, or isolation of the mumps virus) or clinically diagnosed cases presenting with acute, unilateral or bilateral tender swelling of the parotid or other salivary glands lasting one day or more, with an epidemiological link to a lab-confirmed case [19].

Data collection

Data were retrieved from case investigation forms of all notified positive mumps cases reported to the Public Health Directorate, Kingdom of Bahrain, from 2012 to 2022.

Data analysis

Data collected for all positive cases included reporting site, reporting date, age, age group, sex, nationality, residential block, governorate, place of study or work, date of symptoms onset, clinical manifestations of mumps including (swelling and duration, fever, cough, tenderness), travel and contact history, and result date. All data were entered in Microsoft Excel and analyzed in SPSS (version 26; IBM Corp., Armonk, NY). Descriptive analysis of quantitative variables was conducted in the form of mean and standard deviation, and descriptive analysis of qualitative variables was in the form of proportion or ratio. The Chi-square test was used to examine the associations between categorical variables, while the Mann-Kendall test was used to assess trends over time. *P*-values less than 0.05 were considered statistically significant.

Ethical consideration

The study followed the guidelines of the Helsinki Declaration and obtained ethical approval from the Health Research Committee of the Ministry of Health, Kingdom of Bahrain (AUPH-2024-H-4). The study utilized routine surveillance collected data, and patient anonymization along with de-identification was performed before data analysis.

## Results

Patients characteristics

Between 2012 and 2022, a total of 258 confirmed cases of mumps were recorded in the Kingdom of Bahrain. Most of these cases were males, accounting for 159 (61.6%) of the total. Most cases were identified in those aged between 15 and 64 years, representing 108 (41.9%) of the cases, closely followed by the 5 to 14 years age group, which accounted for 86 (33.3%) of the patients. Regarding nationality, 116 (45%) of the patients were Bahraini, and 142 (55%) were non-Bahraini. School-associated cases constituted a significant portion, with 67 (26%) of the cases reporting symptoms while being in school environments. The most common reported symptom was swelling of the parotid gland, observed in 255 (98.8%) patients. Additionally, 242 (93.8%) of the patients experienced parotid gland tenderness, 155 (60.1%) had a fever, and 70 (27.1%) patients reported having a cough. Throughout their illness, 9 (3.5%) of the patients were hospitalized. A travel history shortly before infection was reported in 60 patients (23.3%), classifying them as imported cases, defined as those who traveled to an endemic area within 21 days before symptom onset, aligning with the virus's incubation period. Further, 16 (6.2%) had an epidemiological link to other cases. Of the 258 confirmed cases, only 64 (24.4%) had documented vaccination history. Patients' characteristics are further detailed in Table 1.

**Table 1:** Mumps-positive patients characteristics, 2012-2022.

Characteristics		Vaccination						
		Unvaccinated		Vaccinated		Total		Chi-square
		Count	Row %	Count	Row %	Count	Column %	*P*-value
Age	0-4	44	72.1%	17	27.9%	61	23.6%	0.000*
	5-14	47	54.7%	39	45.3%	86	33.3%	
	15-64	101	93.5%	7	6.5%	108	41.9%	
	>65	3	100.0%	0	0.0%	3	1.2%	
	Total	195	75.6%	63	24.4%	258	100.0%	
Sex	Male	119	74.8%	40	25.2%	159	61.6%	0.726
	Female	76	76.8%	23	23.2%	99	38.4%	
	Total	195	75.6%	63	24.4%	258	100.0%	
Nationality	Non-Bahraini	131	92.3%	11	7.7%	142	55.0%	0.000*
	Bahraini	64	55.2%	52	44.8%	116	45.0%	
	Total	195	75.6%	63	24.4%	258	100.0%	
School case	No	153	80.1%	38	19.9%	191	74.0%	0.004*
	Yes	42	62.7%	25	37.3%	67	26.0%	
	Total	195	75.6%	63	24.4%	258	100.0%	
Swelling	No	3	100.0%	0	0.0%	3	1.2%	0.322
	Yes	222	75.3%	63	24.7%	255	98.8%	
	Total	195	75.6%	63	24.4%	258	100.0%	
Fever	No	73	70.9%	30	29.1%	103	39.9%	0.151
	Yes	122	78.7%	33	21.3%	155	60.1%	
	Total	195	75.6%	63	24.4%	258	100.0%	
Cough	No	142	75.5%	46	24.5%	188	72.9%	0.976
	2	53	75.7%	17	24.3%	70	27.1%	
	Total	195	75.6%	63	24.4%	258	100.0%	
Tenderness	No	13	81.3%	3	18.8%	16	6.2%	0.586
	Yes	182	75.2%	60	24.8%	242	93.8%	
	Total	195	75.6%	63	24.4%	258	100.0%	
Admission	No	188	75.5%	61	24.5%	249	96.5%	0.876
	Yes	7	77.8%	2	22.2%	9	3.5%	
	Total	195	75.6%	63	24.4%	258	100.0%	
Travel	No	140	70.7%	58	29.3%	198	76.7%	0.001*
	Yes	55	91.7%	5	8.3%	60	23.3%	
	Total	195	75.6%	63	24.4%	258	100.0%	
Contact	No	185	76.4%	57	23.6%	242	93.8%	0.209
	Yes	10	62.5%	6	37.5%	16	6.2%	
	Total	195	75.6%	63	24.4%	258	100.0%	

Several factors were significantly associated with being unvaccinated against mumps among the confirmed cases. Notably, non-Bahraini nationals were significantly more likely to be unvaccinated (*P*-value < 0.001). Similarly, cases linked with school outbreaks are associated with being unvaccinated (*P*-value = 0.004). Additionally, individuals with a history of travel before the onset of the infection were found to be more frequently unvaccinated (*P*-value = 0.001) (Table 1). Most of the unvaccinated patients were those aged 15 to 64 (101, 93.5%), followed by those aged 5 to 14 years old (47, 54.7%) (Table 1).

Distribution of cases over time

From 2012 to 2022, the incidence of mumps in the Kingdom of Bahrain showed an overall declining trend (*P*-value = 0.023), with some fluctuations.

In 2012, the number of reported cases was low, but a peak was observed in September with 8 (3.1%) cases. The year 2013 marked an increase, recording the highest number of cases during the study period with 50 (19.4%) cases and an incidence rate of 4 cases per 100,000 individuals. During that year, a surge occurred in June, accounting for 18 cases (6.9%).

**Figure 1:**
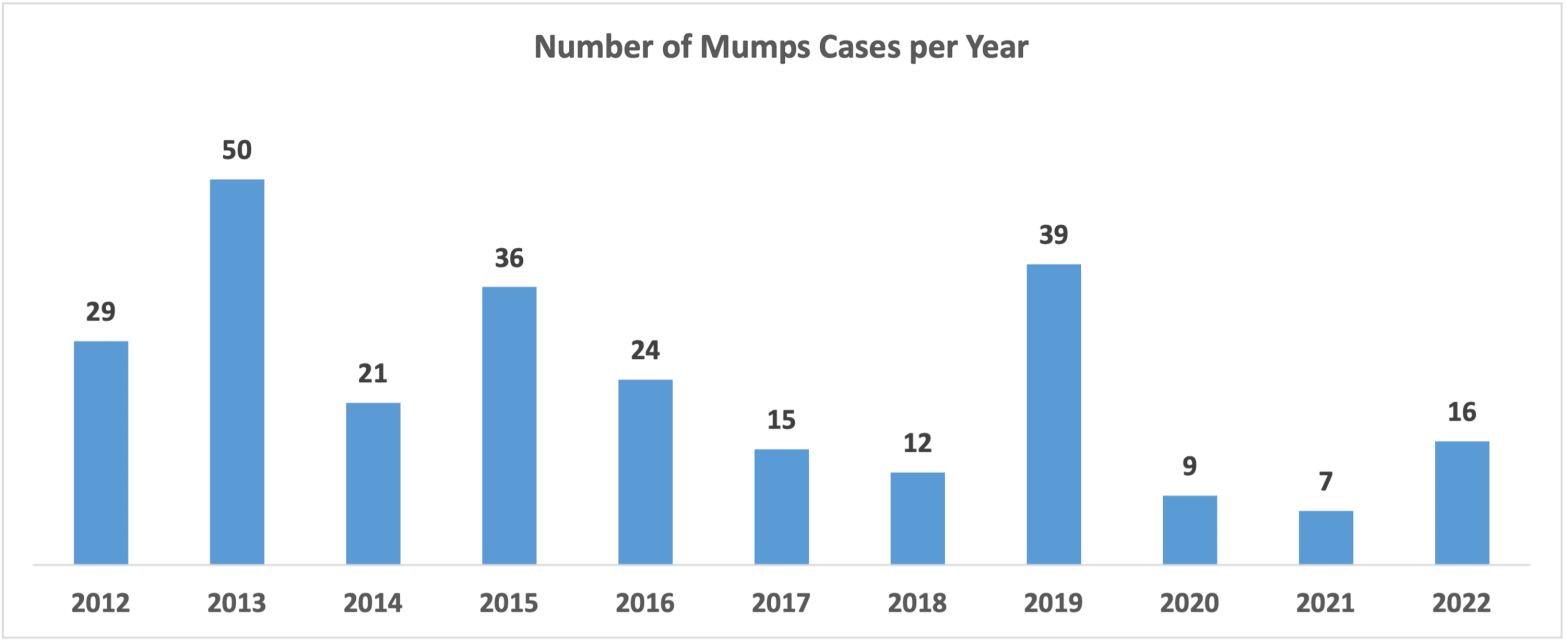
Mumps Cases In Bahrain for the period 2012-2022

**Figure 2:**
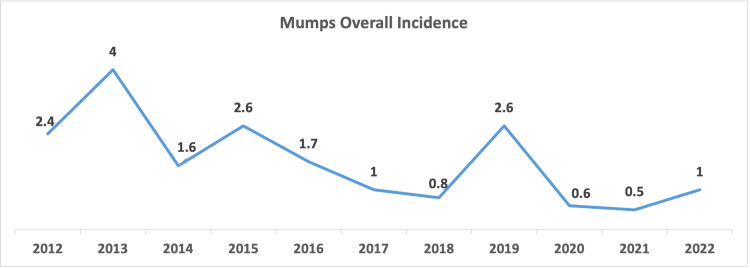
Mumps Overall Incidence (per 100,000 population) in Bahrain for the period 2012-2022

**Figure 3:**
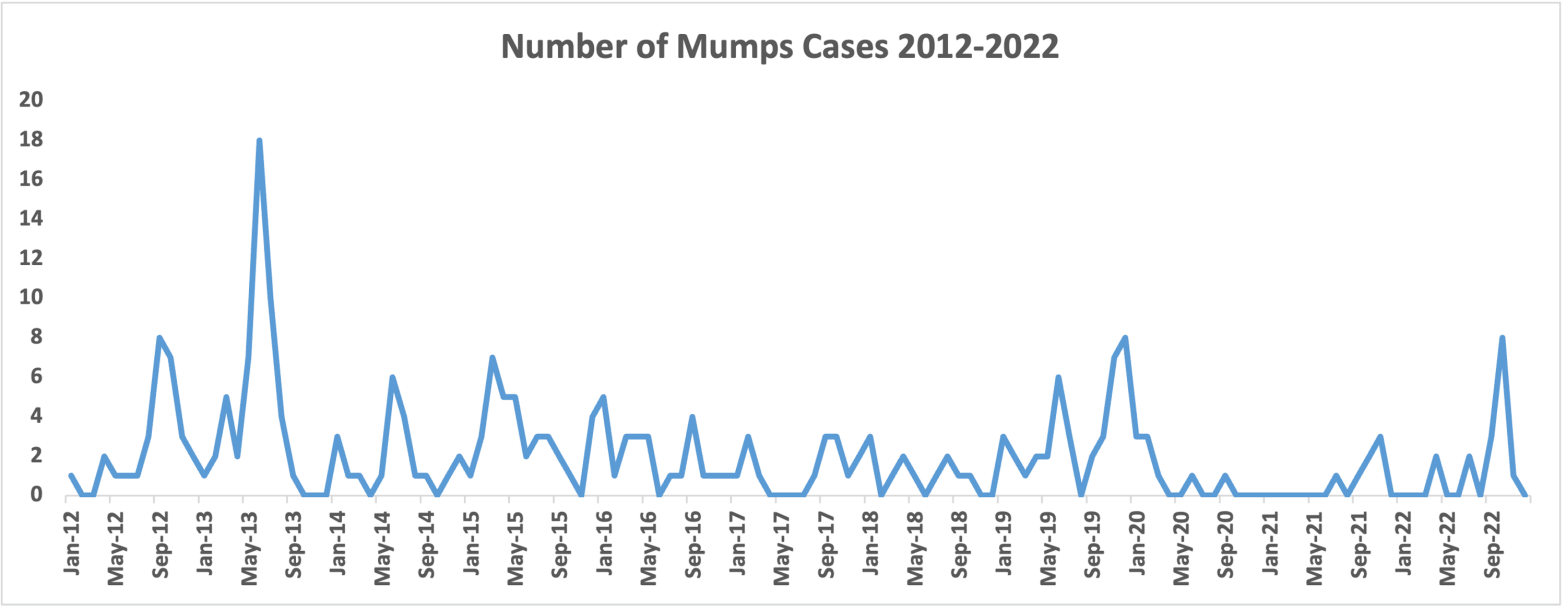
Number of Mumps cases per month and year for the period 2012-2022

In 2014, there was a reduction in cases compared to the previous year. However, 2015 experienced a rise, with 36 (14%) cases and an incidence rate of 2.6 cases per 100,000. The years following saw a general decline in mumps cases. In 2019, a resurgence occurred with 39 cases (15.1%) and an incidence rate of 2.6 cases per 100,000, with a peak in December recording 8 cases (3.1%). The years 2020 and 2021 registered the lowest incidence rates of the study period, with 0.5 and 0.6 cases per 100,000 individuals, respectively. In 2022, the number of cases remained low but noted a peak in October with 8 cases (3.1%). The distributions of cases through the years are presented in Figures 1-3.

**Figure 4:**
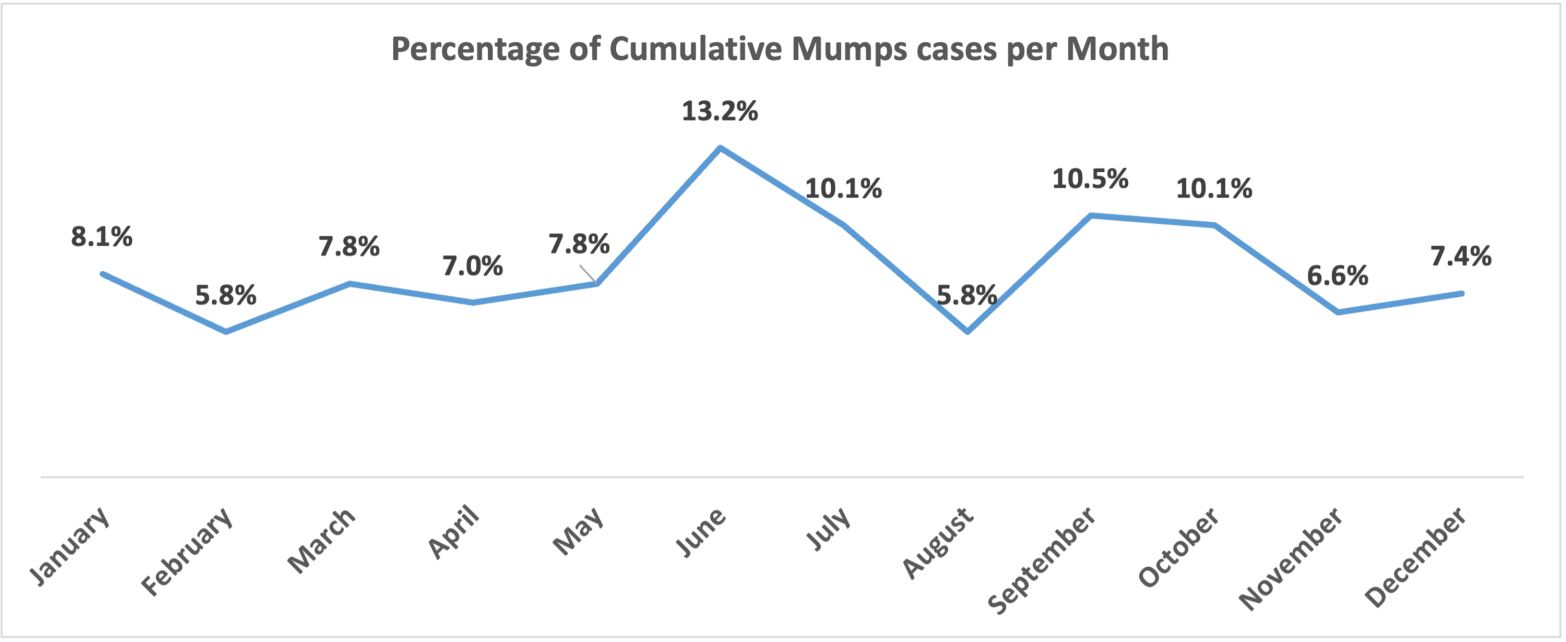
Percentage of cumulative mumps cases per month over the study period (2012-2022).

While no distinct seasonal pattern was evident, two periods showed increased case numbers: from June to July, contributing 13.2% and 10.1% of cases, respectively, and from September to October, accounting for 10.5% and 10.1% of the overall cumulative cases, as presented in Figure 4.

Age-specific incidence

**Figure 5:**
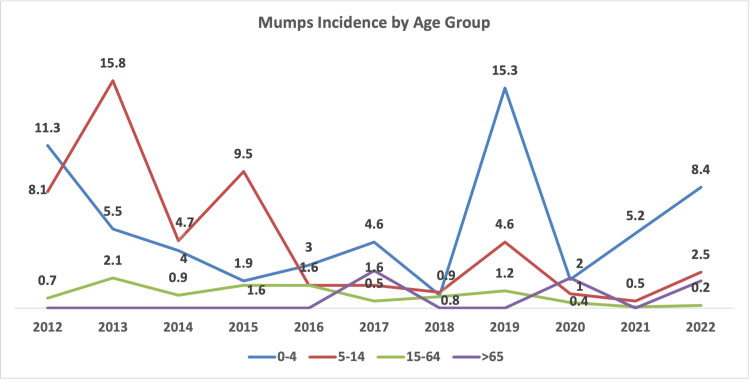
Mumps age-specific incidence (per 100,000) per year for the period 2012-2022.

From 2012 to 2022, mumps incidence varied, with the 0 to 4 years age group recording the highest rate in most years, except between 2013 and 2015, when the highest rates were observed in the 5 to 14 years age group. In 2012, children aged 0 to 4 years had the highest rate, with 11.3 cases per 100,000, followed by the 5 to 14 years age group with 8.1 cases per 100,000. From 2013 to 2015, the highest incidence was seen in the 5 to 14 years age group, ranging from 15.8 to 9.5 cases per 100,000. Meanwhile, the 0 to 4 years age group saw a decline, recording 3 and 1.6 cases per 100,000, respectively (Figure 5).

In 2016, the 0 to 4 years age group again had the highest incidence, followed by the 5 to 14 years and 15 to 64 years age groups, all with rates of 3, 1.6, and 1.6 cases per 100,000, respectively. In 2017, the 0 to 4 years age group remained the most affected, with 4.6 cases per 100,000, followed by those aged 65 and above at 1.6 cases per 100,000. By 2018, the incidence was nearly the same for both the 0 to 4 years and the 5 to 14 years age groups, with 0.9 and 0.8 cases per 100,000, respectively (Figure 5).

In 2019, the 0 to 4 years age group recorded its highest incidence for the entire period, at 15.3 cases per 100,000. In 2020, the 0 to 4 years and the 65+ age groups both had an incidence rate of 2 cases per 100,000. In 2021 and 2022, the 0 to 4 years age group again led with increasing rates, from 2 to 5.2 and then 8.4 cases per 100,000, respectively (Figure 5).

## Discussion

The study provides an overview of the mumps surveillance data in the Kingdom of Bahrain from 2012 to 2022. Most of the reported cases were males (159, 61.6%); this was very similar to the male-to-female ratio reported in a seven-year surveillance study in the United States [20]. However, a study in the United Kingdom reported a lower percentage of males, around 51% [8]. However, both studies reported male predominance.

Almost all patients presented with parotid gland swelling (255, 98.8%) and 242 (93.8%) with gland tenderness. This was similar to the rates reported in the literature reaching over 90% [1,20,21]. Out of all confirmed cases, 9 (3.5%) patients required hospitalization. The hospitalization rate was comparable to rates reported globally: in Australia, the hospitalization rate was 4%, and in the United States, the rate was slightly higher at 6% [9,20]. However, in 2022, the European Center for Disease Prevention and Control (ECDC) reported a higher hospitalization rate of 9% [22].

Only 24% of the patients had documented vaccination history, defined as official records confirming receipt of two doses of the mumps vaccine, which is administered as part of the MMR vaccine at 12 and 18 months of age [17]. This definition may have contributed to an underestimation of the actual vaccination rate among confirmed cases. Globally, there has been an increase in reports of mumps outbreaks among vaccinated individuals. For example, in a study conducted in Australia, the vaccination rate among reported mumps cases was around 62% [9]. Another study from the United States reported a vaccination rate of 71%, although this was defined as receiving only one dose of the vaccine [20]. In Ireland, while 72% had received two doses, only 32% of patients had documented vaccination status, possibly leading to an overestimation [23].

Approximately 24% of the confirmed cases were classified as imported, defined as individuals with a recent travel history to an endemic area within 21 days before symptom onset. Although this figure is higher than the 3.4% of imported cases in the United States, it is noteworthy that 37% of US cases had an unknown origin [20]. Pre-COVID-19 pandemic, young adults were frequently the most affected, primarily due to outbreaks in educational settings, as documented in Canada, the United States, and Korea [11,20,24]. In the United States, a peak incidence was observed in the 18-24 years age group from 2018 to 2020, while from 2021 to 2023, it was highest among children aged 1-4 years [20]. In both Australia's 2015-2016 outbreak and Ireland's 2018-2020 outbreak, the highest incidence was among those aged 15-19 years [9,23].

As with many studies using surveillance data, this study had its limitations. Although we had a strong surveillance system in Bahrain, the underreporting of cases cannot be fully excluded. Low clinical suspicion in fully vaccinated patients and incomplete data for some variables further impacted results. Additionally, the surveillance period from 2020 to 2022 was affected by the COVID-19 pandemic, potentially leading to a decline in data quality. The effect of social distancing in the transmission of the disease can not be ignored during the pandemic period. Furthermore, the influence of confounders in the relationship between the assessed factors and vaccination status cannot be ruled out. However, the study had several strengths. First, to our knowledge, it is the first study to examine mumps epidemiology in Bahrain, providing valuable baseline data to enhance future surveillance and public health efforts. Second, the study included more than 10 years, allowing for a comprehensive analysis of long-term trends and shifts in mumps incidence patterns. Third, the study utilized surveillance data from all healthcare facilities in Bahrain, including both public and private sectors, making the findings more inclusive and representative of the entire population because mumps notification to public health is mandated by public health law. Also, reliance on routinely collected data helped minimize recall bias among patients.

The findings of this study highlight the critical importance of complete vaccination, especially among school children and non-Bahraini residents. The higher number of unvaccinated cases in these groups indicates gaps in immunization coverage that need to be addressed. Strengthening the enforcement of vaccination requirements before school entry could help prevent future mumps outbreaks. Focusing public health efforts on increasing vaccine uptake among these populations and maintaining high overall vaccination coverage is essential to reduce the incidence of mumps in the Kingdom of Bahrain.

## Conclusions

A total of 258 confirmed mumps cases were reported in Bahrain between 2012 and 2022, with the majority being male. Nearly all patients presented with parotid gland swelling and tenderness. Only 24% had documented vaccination history. The highest number of cases occurred in 2013, 2019, and 2015. In 2012, the peak incidence was among children aged 0-4 years, shifting to the 5-14 age group from 2013 to 2015. However, from 2016 to 2022, the highest incidence returned to the 0-4 age group. Overall, mumps incidence rates have been decreasing in Bahrain.
